# Modular bioreactor for primary human hepatocyte culture: Medium flow stimulates expression and activity of detoxification genes

**DOI:** 10.1002/biot.201000326

**Published:** 2011-05

**Authors:** Bruna Vinci, Cédric Duret, Sylvie Klieber, Sabine Gerbal-Chaloin, Antonio Sa-Cunha, Sylvain Laporte, Bertrand Suc, Patrick Maurel, Arti Ahluwalia, Martine Daujat-Chavanieu

**Affiliations:** 1 Centro Interdipartimentale di Ricerca “E. Piaggio”, Faculty of Engineering, University of PisaPisa, Italy; 2 InsermU632, Montpellier, France; 3 Université Montpellier1UMR-S632, Montpellier, France; 4 CHU Montpellier, Institut de Recherche en BiothérapieHôpital Saint Eloi, Montpellier, France; 5 Sanofi-Aventis, Recherche et dévelopement, Département DMPK-SMontpellier, France; 6 CHU Bordeaux, Service de Chirurgie Digestive, Hôpital Haut-LévèquePessac, France; 7 CHU Nîmes, Chirurgie Viscérale et Digestive, Hôpital CaremauNîmes, France; 8 CHU Toulouse, Service de Chirurgie Digestive et de TransplantationToulouse, France

**Keywords:** Bioreactor, Detoxification, Human hepatocyte, Shear stress, Xenosensors

## Abstract

Down-regulation of detoxification genes, notably cytochrome P450 (CYPs), in primary hepatocyte cultures is a long-standing and major concern. We evaluated the influence of medium flow in this model. Hepatocytes isolated from 12 different liver donors were cultured either in a multichamber modular bioreactor (MCmB, flow rate 250–500 μL/min) or under standard/static conditions, and the expression of 32 genes, enzyme activities and biological parameters were measured 7–21 days later. mRNA expression of genes involved in xenobiotic/drug metabolism and transport, including CYP1A1, 1A2, 2B6, 2C9, 3A4 (and activities for some of them), UDP-glucuronosyltransferase (UGT) 1A1, UGT2B4, UGT2B7, glutathione S-transferase (GSTα), and multidrug resistance protein 1 (MDR1) and MRP2, were specifically up-regulated by medium flow as compared with static controls in all cultures tested. In 2-week-old cultures, expression of detoxification genes reached levels close to or higher than those measured in freshly isolated hepatocytes. In contrast, CYP2D6 and most of other tested genes were not affected by medium flow. We conclude that medium flow specifically interferes with, and up-regulates, the activity of xenosensors and/or the expression of detoxification genes in primary human hepatocytes. Down-regulation of detoxification genes in conventional (static) cultures is therefore partly a consequence of the absence of medium circulation.

## 1 Introduction

Primary cultures of normal adult human hepatocytes are considered as the gold standard for investigating drug/xenobiotic metabolism, toxicity and side effects [1]. However, a major concern while using these cultures is the early down-regulation of many detoxification genes including cytochrome P450 (CYPs), conjugation enzymes and xenobiotic membrane transporters [2]. Co-cultivation of hepatocytes with nonparenchymal cells [3], chromatin remodeling agents (such as dimethyl-sulfoxide [4] and trichostatin A [5]), specific culture media [6], matrigel [7], collagen sandwich configuration [8], and use of inducer cocktails [9] have been shown to improve the maintenance of hepa-tocyte phenotype and detoxification function [1]. However, the huge drop in CYP expression after cell plating remains a major problem. This is likely due to the fact that some physiological stimuli are not replicated in standard culture conditions.

Previous studies on endothelial and hepatic cell cultures have shown that some CYP genes are induced through blood flow-mediated shear stress [10–21]. However, the viability of hepatocyte cultures has been reported to be compromised under high shear as compared with static controls [22, 23]. A low shear stress generic bioreactor system [multichamber modular bioreactor (MCmB)] has therefore been designed and patented [24]. This system has been tested successfully in connected cultures of human umbilical vein endothelial cells and rodent hepatocytes, as well as with HepG2 cells [25].

During the last decade we have developed long-term primary cultures of human hepatocytes [6, 26–28]. Here we evaluate the influence of culture medium flow on hepatocyte phenotypic markers in the MCmB. No significant change was observed with α1-antitrypsin, blood coagulation factors V and VII, or xenosensors such as aryl hydrocarbon receptor (AhR), pregnane X receptor (PXR), and constitutive androstane receptor (CAR), although a non-significant decrease was reproducibly observed with albumin and carbamoyl phosphate synthase 1 (CPS1) under dynamic conditions. In contrast, medium flow significantly stimulated the expression of detoxification genes, including CYP 1-3, UDP-glucuronosyltransferases (UGTs; 1A1, 2Bs), GSTα and transporters such as mul-tidrug resistance protein 1 (MDR1) and MRP2, so that expression levels higher than or close to those measured in freshly isolated hepatocytes were reached after several weeks. Medium flow appears therefore to up-regulate the detoxification function in primary human hepatocytes.

## 2 Materials and methods

### 2.1 Primary cultures of human hepatocytes

Hepatocytes were isolated either from liver lobec-tomies or from livers of organ donors unsuitable for transplantation (Table 1) under approval of Montpellier University and the National Ethics Committee (last renewal, October 2008), and cultured as described [26].

**Table 1:** Clinical characteristics of liver donorsa)

Liver	Sex	Age	Pathology	Experiments
FT270	M	57	Metastasis of colic cancer	qRT-PCR, FIH
FT297	M	82	Metastasis of rectum carcinoma	MCmB, qRT-PCR, FIH
FT298	LJ_	53	Cholangiocarcinoma	MCmB, qRT-PCR, FIH
FH301	F	28	Organ donor	MCmB, qRT-PCR
FT300	M	72	Metastasis of rectum carcinoma	qRT-PCR, FIH
FH302	F	73	Organ donor	qRT-PCR, FIH
FT303	F	51	Metastasis of endocrine tumor	qRT-PCR, FIH
FT306	M	61	Metastasis of gastric carcinoma	MCmB, drug metabolism
FT309	M	67	Cholangiocarcinoma	MCmB, drug metabolism
FT315	M	60	Metastasis of colon carcinoma	MCmB, flux effect, flow rate, conditioned medium
FT316	F	51	Angiocholitis	MCmB, flux effect, flow rate, conditioned medium
FT317	M	74	Metastasis of rectum carcinoma	MCmB, flux effect, flow rate, conditioned medium

a) qRT-PCR, quantitative RT-PCR analysis; FIH, freshly isolated hepatocytes, MCmB, multichamber modular bioreactor.

### 2.2 Bioreactor and cell cultures

**Figure 1:**
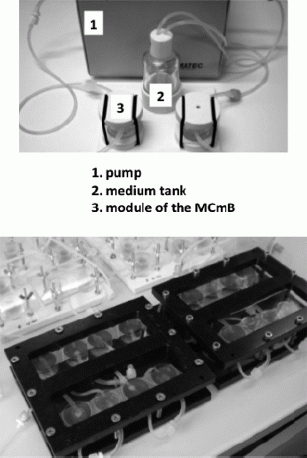
Multichamber modular bioreactor. The whole system is placed in a cell culture incubator.

**Figure 2:**
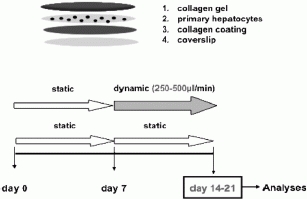
Experimental protocol of the studies.

The MCmB, fabricated at the University of Pisa, consists of silicone culture chambers (similar shape and dimensions as those of 24-well plates) connected by culture medium flow, so that cell-cell interaction between chambers is mediated by soluble molecules/proteins as in the body. The priming volume of the bioreactor chambers is 3 mL and the total volume of each chamber is 2 mL. At a flow rate of 500 μL/min, the perfusion time of each bioreactor is 4 min. This flow rate corresponds to a wall shear stress of 10^−5^ Pa (5 × 10^−6^ dyne/cm^2^) at the cell surface, which is close to the levels of shear predicted by models of interstitial flow in soft tissues [29]. The MCmB was inoculated with adult human hepatocytes in long-term culture conditions [26]. Hepatocytes were plated on glass coverslips (1.2 cm diameter, cell density 1.7 × 10^5^ cell/cm^2^) coated with collagen (BD, Pont La Chaix, France), and cells were overlaid with collagen in a sandwich configuration [8]. Experiments were performed in two steps. First (days 1–7), coverslips were placed in petri dishes under static conditions without medium change. Second (days 8–14 or longer) coverslips were placed either: (i) in MCmB chambers, medium was renewed and cells were submitted to a flow rate of 250–500 μL/min, without further medium change (dynamic conditions), or (ii) in a new petri dish, medium was renewed and culture was continued under static conditions without further medium change (static conditions). The ratio of cell number to medium volume was 1.7 × 10^5^ cells/3 mL in all experiments (dynamic and static conditions). In parallel experiments, hepatocytes were cultured under standard conditions on collagen or in collagen sandwich (0.8 × 10^6^ cells/mL) for 14 days or longer. Experimental design and protocols are shown in Figs. 1 and 2.

### 2.3 Quantitative RT-PCR

Total cellular RNA was extracted using TRIzol reagent (Invitrogen, Cergy Pontoise, France). mRNA expression was evaluated by quantitative RT-PCR using Roche LightCycler (Roche Diagnostics, Meylan, France). The following program was used: one step at 95°C for 10 min, 55 cycles of de-naturation at 95°C for 15 s, annealing at 65°C for 15 s and elongation at 72°C for 15 s. Amplification specificity/quality was assessed by analyzing product melting curves. Relative quantification was calculated with the Pfaffl method [30] and normalized to ribosomal protein, large, P0 (RPLP0) RNA expression. Sequences of primers, designed from different exons to avoid false positives due to DNA contamination, are shown in the Supporting information, Table SI.1.

### 2.4 Drug metabolism assays

After 7 days in MCmB (medium flow 250 μL/min), coverslips were placed in wells of 24-well plates, 180 μL fresh media was added and drug metabolism experiments were performed with 20 μM dextromethorphan (CYP2D6), 5 μM midazolam (CYP3A4 and UGT2B4/7) or 5 μM tolbutamide (CYP2C9). At 2, 4, 8 and 24 h, 400 μL acetoni-trile/water (30:10) was added to each well and extracellular medium and cell homogenate were mixed and submitted to analysis for substrate and metabolites by LC/MS-MS using MassLynx 4.0 Software (Waters-Micromass, Milford, MA, USA) as described [31].

### 2.5 Albumin secretion

Albumin secretion was measured using an enzyme-linked immunosorbent assay (Bethyl Laboratories, Montgomery, TX, USA). Non-linear curves were fitted using MARS data analysis software (BMG LABTECH GmbH).

### 2.6 Urea secretion

Urea secretion was measured using Quanti-Chrom™ Urea Assay Kit (Gentaur, Paris, France), under manufacturer recommendations.

### 2.7 Statistical analysis

Each experiment was carried out in duplicate per liver as listed in Table 1. The data are reported as means and SEM for up to six livers per experiment. Significance testing was based on analysis of variance and the student's *t*-test, a *p* value of <0.05 being considered as significant.

## 3 Results

### 3.1 Effect of culture medium flow on mRNA expression of genes involved in endogenous metabolism and hepatic function

**Figure 3:**
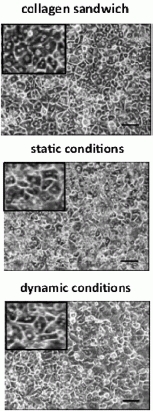
Phase-contrast microscope examination of hepatocytes (FT297) in collagen sandwich configuration under standard conditions (top), static conditions (middle) and dynamic conditions (250μL/min, bottom), after 14 days in culture. Bar = 100 μm.

Hepatocytes were first cultured for 7 days under static conditions to allow the down-regulation of genes and then submitted to dynamic conditions in the MCmB for another 7 days (or longer). In the meantime, control cells were cultured under static conditions (14 days or longer). The flow rate used is the optimum balance between low shear stress and high oxygenation [24], and corresponds to a perfu-sion time of around 3–5 min per bioreactor, similar to the average perfusion time in human liver. On microscope examination, hepatocytes exhibited typical and similar aspects under both conditions (Fig. 3). As reported in supporting information, Table SI.2, on average no significant change was observed in albumin, α1-antitrypsin (AAT), Factor V, Factor VII, CPS1, glucose-6 phosphatase (G6P), phosphoenol pyruvate carboxykinase 1 (PEPCK1), glucokinase (GK), pyruvate kinase (PK-L), apolipoprotein (Apo) F, ApoH, hepatocyte nuclear factor 4α (HNF4α) and CAAT/enhancer binding protein α (C/EBPα) mRNA expression or in the production of albumin and urea (Supporting information, Table SI.3) under dynamic (250 μL/min) versus static conditions.

### 3.2 Detoxification genes are induced by the culture medium flow in human hepatocytes

As reported in Table 2, CYP1A1, CYP1A2, CYP2B6, CYP2C9, CYP3A4, UGT1A1, UGT2B4, UGT2B7, GSTα, MDR1 and MRP2 mRNA levels were induced under dynamic versus all other culture conditions. It must be emphasized here that same observations were made on fresh hepatocyte cultures (between days 1 and 5 post-plating, not shown). CYP1A1, CYP2B6, UGT1A1, MDR1 and MRP2 mRNA levels were even greater in dynamic conditions than in freshly isolated hepatocytes (FIH). Interestingly, expression of CYP2D6, Na(+)/tauro-cholate transport protein (NTCP) and organic anion transport protein 1B3 (OATP1B3) mRNAs was not affected by the flow. Finally, glucocorticoid receptor, AhR nuclear translocator, and PXR were expressed in cultured hepatocytes at a level close to that observed in FIH, and their expression was not sensitive to medium flow when compared with normal culture conditions, while AhR expression was slightly increased. CAR was expressed at very low levels under all conditions.

**Table 2:** Effect of medium flow on expression of detoxification genesa)

Gene	Dynamicb)	Static**	Sandwichb)	Collagenb)	FIH
CYP1A1	759.4±550.3	29.2±26.4**	39.7±39.6**	32±30**	100±73.9**
CYP1A2	24.4±8.1	2.2±1.4**	3.8±2.5**	3.0±3.0**	100±64.5*
CYP2B6	230.1±183.1	1.8±3.3*	1.0±0.9*	1.6±2.4*	100±53*
CYP2C9	17.8±7.4	4.4±1.9**	9.0±7.0*	8.1±3.5*	100±75.5*
CYP2D6	29.4±26.5	34.1±32.6	45.7±25.6	33.8±28.4	100±29.7**
CYP3A4	125.8±98.1	2.6±1.9*	1.6±0.7*	2.3±1.8*	100±100.1
UGT1A1	227.9±118.3	55.8±25.8*	99.6±31.4*	62.3±33.4*	100±63.6**
UGT2B4	96.2±50.5	29±17.5*	77.1±29.4	44.5±33.3*	100±45**
UGT2B7	26.3±7.6	8.1±6.5**	33.9±26	18.3±6.9	100±87**
GSTA1	44.5±16.9	14.4±5.7**	18.9±7.3**	18.1±7.9**	100±48.7*
NTCP	1.9±1.4	2.4±1.3**	9.0±2.4**	14.1±3.6**	100±71.1**
OATP1B3	1.9±1.0	1.5±1.1	4.6±2.8	3.1±2.2*	100±52.8**
MDR1	284.3±60.3	86.9±10.3**	89.3±32**	97.9±32.7**	100±54.1**
MRP2	1387.5±863.1	333.4±271.9*	344.6±149.8*	442.6±246.8*	100±134*
AhR	53.4±19.2	26.5±7.6**	52.4±24.4	54.8±42.6	100±84.8
ARNT	34.6±8.3	35.5±5.4	57.5±11.1**	42.7±10.4	100±51**
CAR	1.9±0.7	1.7±1.1	3.4±2.7	1.8±0.6	100±54**
PXR	95.8±21.9	86.4±16.9	112.0±36.5	99.3±29.5	100±53.9
GR	44.9±7.3	38.2±11.7	45.7±10.6	56.8±16.1	100±32.7

a) ARNT, AhR nuclear translocator; GR, glucocorticoid receptor; NTCP, Na(+)/taurocholate transport protein; OATP1B3, organic anion transport protein 1B3.

b) Dynamic and Static: see Fig. 2. Sandwich and Collagen: standard conditions in collagen sandwich configuration or on collagen (0.8 × 10_6_ cells/mL).

See Materials and methods for details.

* *p*<0.05.

** *p*<0.005 with respect to gene expression in dynamic conditions (*n*=6).

Rates of tolbutamide 4-hydroxylation (CYP2C9), dextromethorphan *O*-demethylation (CYP2D6) and midazolam 1-hydroxylation (CYP3A4) and *O*-glucuronidation (UGT2B4/7) [32] are reported in Table 3. Interestingly and consistent with data reported in Table 2, tolbutamide 4-hydroxylation and midazolam 1-hydroxylation and *O*-glucuronidation were induced in two independent cultures (FT306 and FT309) under dynamic conditions and reached levels close to the range observed for FIH, irrespective of the fact that these activities are widely variable from one culture to another. In contrast, dextromethorphan *O*-demethylation was not affected by medium flow. Note that this activity was much lower than the mean activity observed in FIH.

**Table 3:** Effect of medium flow on CYP-mediated monoxygenase activities

Conditions	Tolbutamide 4-hydroxylationa)	Dextromethorphan *O*-demethylationa)	Midazolam 1-hydroxylationa)	Midazolam *O*-Glucuroa)
FT306				
Static	0.012	0.034	0.013	0.016
Dynamic	0.021	0.02	0.10	0.19
	(1.75)b)	(0.59)	(7.7)	(11.8)
FT309				
Static	0.002	0.022	0.02	0.014
Dynamic	0.042	0.014	0.45	1.01
	(21)	(0.64)	(22.5)	(72)
FIH (mean)c)	0.057±0.035	0.84±0.67	0.83±0.70	0.31±0.26

a) Activities are in nmol/h/10^6^ cells. Tolbutamide 4-hydroxylation: CYP2C9; dextromethorphan *O*-demethylation: CYP2D6; midazolam 1-hydroxylation: CYP3A4; midazolam *O*-glucuronidation: UGT2B4/7.

b) In parenthesis: the fold induction between dynamic versus static conditions.

c) *n*=78 for tolbutamide 4-hydroxylation and midazolam 1-hydroxylation, and *n*=96 for dextromethorphan *O*-demethylation.

### 3.3 Detoxification gene expression is sensitive to culture medium flow rate and duration of exposure

**Figure 4:**
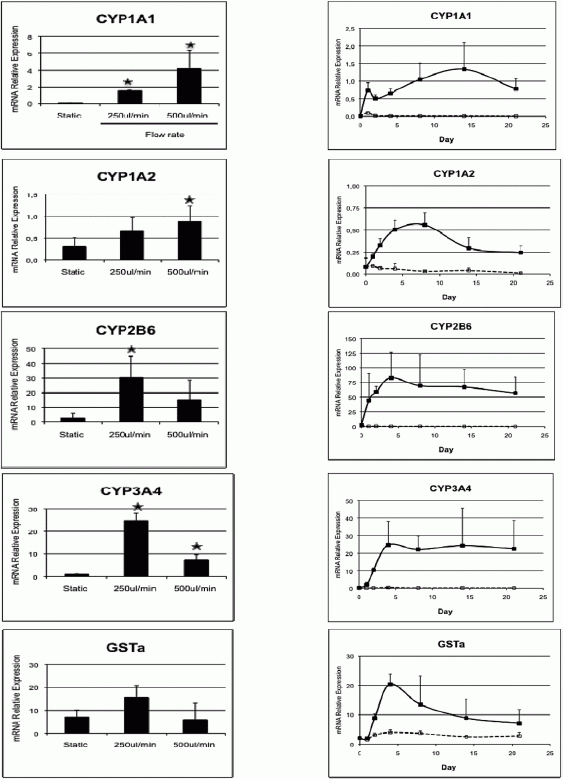
Effect of flow rate and duration of exposure to the flow on gene expression. Hepatocytes were cultured as indicated in Fig. 2. Left panels (FT316): effect of rate of medium flow at day 7 on mRNA expression normalized with respect to ribosomal protein, large, P0 (RPLP0) mRNA (arbitrary units). Right panels (FT317): effect of time of exposure to flow (250 μL/min). Black squares: dynamic; white squares: static conditions. *p<0.05 with respect to static conditions; *p*<0.01 for CYP1As.

Next, the influence of both the rate of medium flow and the duration of exposure to the flow was evaluated on responsive genes. For this purpose, 12 bioreactor chambers were placed in series so that two samples per time point could be analyzed over 21 days. The results are shown in Fig. 4. Different expression patterns were observed. CYP1A1 and CYP1A2 mRNA expression increased with flow rate and reached a maximum after 15 and 7 days, respectively (*p*<0.01), while CYP2B6, CYP3A4 and GSTα mRNA expression reached a maximum at 250 μL/min and decreased thereafter, maximum expression requiring at least 4 days of exposure to flow.

### 3.4 Effect of bioreactor conditioned media on detoxification gene expression

**Figure 5:**
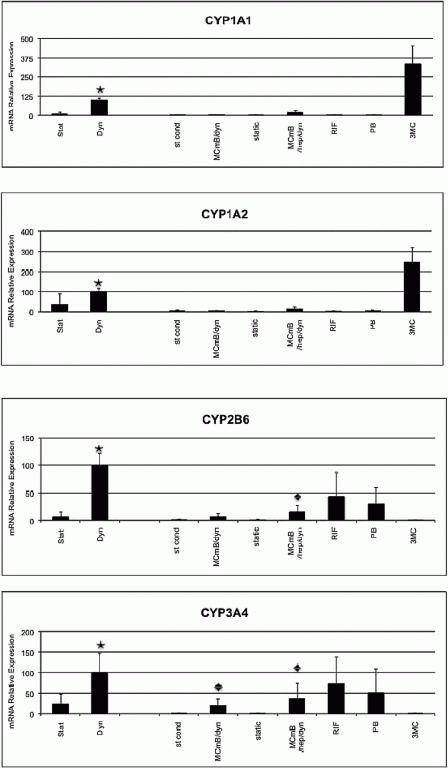
Effect of flow, conditioned medium and xenobiotics on gene expression. Hepatocytes (FT315, 316, 317) were cultured as indicated in Fig. 2 and analysis of gene expression was carried out at day 14. Stat (static conditions) and Dyn (dynamic conditions, flow 250 μL/min). From left to right for standard conditions (std cond) to 3MC: 1-week-old hepatocytes were untreated (std cond) or treated for 24 h with: conditioned media including medium circulated (250 μL/min) in the bioreactor for 7 days in the absence (MCmB/dyn) or presence (MCmB/hep/dyn) of hepatocytes, medium maintained in culture dishes in the presence of hepatocytes for 7 days (static), RIF (25 μM), PB (1 mM) or 3MC (25 μM). For each individual gene, the average ratio of mRNA expression to RPLP0 mRNA (arbitrary units, *n*=3) is presented. *p<0.05 with respect to Stat. Black diamond *p*<0.05 with respect to standard (st) conditions.

Detoxification genes that are flow-responsive are inducible by xenobiotics through AhR, CAR and/or PXR xenosensors [33–36]. We therefore suspected that activators or agonists of xenosensor are released from, or produced in the MCmB. To check this possibility, we prepared culture medium samples that had been:( i) circulated (250 μL/min) in MCmB without hepatocyte (MCmB/dyn), (ii) in the presence of hepatocytes (MCmB/hep/dyn), (iii) or maintained in culture dishes in the presence of hepatocytes (static), for 7 days. Hepatocytes were then treated for 24 h with these medium samples or prototypical inducers [3-methycholanthrene (3MC),rifampicin (RIF) or phenobarbital (PB)] under standard conditions and CYP1A1/2, 2B6 and 3A4 mRNA levels were measured (Fig. 5). Hepatocytes responded specifically to prototypical inducers as expected (3MC, RIF or PB versus standard conditions). When compared with dynamic conditions (Dyn), CYP1A1 and 1A2 mRNA levels were two to three times greater in 3MC-treated cells, while CYP2B6 and CYP3A4 mRNA levels were approximately 60% or less in RIF- or PB-treated cells (3MC, RIF or PB versus Dyn). CYP mRNA levels observed in conditioned media-treated cells were approximately 10–40% of those observed in dynamic conditions (MCmB/dyn and MCmB/hep/dyn versus Dyn). These effects were always greater when conditioning was made in the presence of hepatocytes (MCmB/hep/dyn versus MCmB/dyn). However, this induction, which was only significant for CYP3A4 and CYP2B6, did not account for the whole increased expression observed under dynamic conditions.

## 4 Discussion

To our knowledge, this is the first demonstration that medium flow increases the expression of several phase I, II and III detoxification genes controlled by AhR, CAR and PXR, in primary human hepatocytes.

Several studies have been carried out previously to investigate the influence of medium flow on arterial and venous endothelial cells. Indeed, these cells are continuously exposed in vivo to high shear stress of 10–30, and 1–7 dyne/cm^2^, respectively [37]. CYP1A1 and CYP 1B 1 mRNA, protein and activity were shown to be increased in a time- and shear-dependent manner in venous endothelial cells cultured in parallel plate flow chambers (shear stress 15–25 dyne/cm^2^) [10, 11]. It was suggested that shear stress produces circulating AhR ligands through CYP1A1 enzyme that activates CYP1 expression. Han et al. [14] reported that shear stress induced both AhR expression through MAPK-de-pendent activation and nuclear translocation, and that AhR antagonist and specific siRNA suppressed CYP1A1 induction mediated by flow. Zhu et al. [21] reported that shear stress induces LXR and CYP27A via a PPARγ-mediated pathway. Transcriptome analysis identified several flow-sensitive genes including zinc finger protein EZF/GKLF, glucocorticoid-induced leucine zipper protein, and Krüppel-like factor [15, 38]. Moreover, SP1 and Ets-1 were shown to be involved in the shear stress-induced expression of plasminogen activator inhibitor-1 in rat hepatocytes [39]. However, in spite of these observations, the molecular mechanism of CYP induction by medium flow in endothelial cells is currently unknown.

Fewer studies have been devoted to hepatic cells. HepG2 cells were shown to respond to hydro-dynamic shear stress by transient increase in AhR-mediated CYP1A1 activity [17, 18], and by increased expression of CYP3A4 and UGT2B7 [20]. An increase in CYP1A1 activity (ethoxyresorufin deethylation) was also observed in rat hepatocyte under dynamic conditions [13, 19]. Our results provide new evidence that flow affects the expression of detoxification genes of phase I, II and III, in primary human hepatocytes.

Several differences should be noted, however, between previous and the current study. First, under our conditions, hepatocytes responded to much smaller shear stress (i.e., 0.5 μdyne/cm^2^ shear stress or 5 μPa) as compared with endothelial cells (1–30 dyne/cm^2^) [10, 11, 14–16, 19, 21, 38]. From the anatomic point of view, and in contrast to endothelial cells, hepatocytes are not expected to be submitted to high shear in the liver because: (i) the blood flow is divided in thousands of sinusoids, (ii) hepatocytes are protected by sinusoid endothelial cells and the space of Disse, and (iii) they are perfused by low velocity interstitial flow. In addition, hepatocytes and endothelial cells derive from different germ layers, i.e., the endoderm and mesoderm, respectively [40]. It is accordingly expected that these cells should exhibit quite different phenotypic traits, especially in response to the flow, which is a major environmental factor for endothelial cells. Moreover, it was recently reported that individual cell types display unique responses to flow in the MCmB, suggesting that shear stress effects are dependent on specialized cell-specific mechanoreceptors [41]. In summary, these various arguments support the findings that hepatocytes and vein/artery endothelial cells respond differently to flow-induced shear stress, not only in terms of the magnitude of the shear that triggers the response, but also in terms of the kinetics of the response to the flow (Fig. 4). Indeed, maximal responses were reached in hepatocytes after 4–15 days depending on genes, while less than 24 hours are necessary with endothelial cells. Second, we observed a modest induction of AhR mRNA, in agreement with others [11, 14], but not of xenosensors CAR and PXR mRNAs in response to the flow (Table 2), and glucocorticoid receptor, which controls CAR and PXR gene expression [42], was not induced either. Third, MCmB/hepatocyte-conditioned medium significantly induced CYP3A4 and CYP2B6 mRNAs to a greater extent than MCmB/without cell-conditioned medium (Fig. 5). This finding suggests that either a xenosensor agonist or an activation process that remains to be identified is generated or triggered by hepatocytes in the MCmB. It is important, however, to emphasize that this contribution, whatever the mechanism, represents less than 40% of the flow-mediated increase in gene expression. Fourth, in addition to the up-regulation of CYP1As known to be regulated by AhR, the flow induced the expression of other genes (including CYP2B6, CYP2C9, CYP3A4, UGT1A1,UGT2B4/7,GSTα,MDR1 and MRP2) that are known to be regulated by CAR and/or PXR [36]. Interestingly, our results show clear differences: (i) on the influence of the rate of flow with a constant increase for CYP1As, whereas a maximum is reached with CYP2B6 and CYP3A4 (Fig. 4), and (ii) on the flow effect versus prototypical agonist effects with a greater induction of CYP2B6/3A4 by flow as compared with prototypical inducers, while the opposite was observed with C YP1As (Fig. 5). Although we have no interpretation for these observations, the different behavior of these two groups of genes is most likely due to the fact that they are primarily regulated by different xenosensors, AhR (CYP1As) versus CAR/PXR (CYP2B6, CYP3A4). These xenosensors are known to exhibit different molecular biology and mechanisms of activation, in addition to being activated by different agonists [33–36]. A different response of their transcription-al activity to the flow should therefore not be surprising. Overall, our data demonstrate that the flow is able to activate different signaling pathways in human hepatocytes.The finding that PXR/CAR-responsive genes are induced by the flow, whereas these xenosensors are not, is not contradictory. Indeed, most of prototypical inducers of PXR/CAR, such as RIF or PB, which are potent inducers of CYP2B6 and CYP3A4 by triggering the transcriptional activity of these xenosensors, have no effect on their expression at both the mRNA and protein levels. Whether flow, be it due to the mechanical stimulus offered by shear or to convection-aided medium turnover in the form of increased oxygen or nutrient supply and catabolite removal [43], or both, interferes with and modulates the transcrip-tional activity of these xenosensors or directly regulates the expression of detoxification genes is unknown and will require further investigations.

Another important point with CYP protein activities that deserves consideration is oxygen concentration. The current data on CYP2D6 suggest that a difference in oxygen concentration between the MCmB versus static cultures cannot explain our results. In contrast to other CYPs analyzed here, CYP2D6 mRNA level is not affected by the flow and this correlates with unchanged specific activity (detromethorphan oxidation) (Tables 2 and 3). A limiting amount of oxygen in static versus MCmB conditions should have been accompanied by a decreased activity in static versus MCmB, which is not the case.

Since non-hepatocyte cells are likely to be present (as contaminant) in long-term cultures of hepatocytes, we suspected that our observations reflect the effect of medium flow on these contaminating cells. However, careful microscope examinations (Fig. 3) revealed no important contamination by such cells. In addition, it is known that non-parenchymal liver cells do not express significant levels of detoxification enzymes in vivo, notably those that are regulated by PXR/CAR such as CYP3A or CYP2 genes [1, 2]. Furthermore, previous results have shown that endothelial cells respond to very high shear stress [10, 11] in comparison to hepatocytes. These arguments suggest that the flow-mediated induction observed here is not related to contaminating non-parenchymal liver cells. The possibility that cell survival can be affected by the flow in the MCmB has been considered but was not supported by microscope examination (Fig. 3). In addition, greater activities or expression levels (Tables 2 and 3) were observed under the MCmB conditions with respect to static conditions. Moreover, results from Tables SI.2 and SI.3 showed that albumin gene expression and protein production (a classical liver phenotypic marker) do not differ between conditions.

In conclusion, submitting primary human hepatocytes to culture medium flow, which mimics the blood flow in the liver, is sufficient to restore the expression of detoxification genes regulated by AhR, CAR and PXR to levels close to, or higher than, those observed in FIH. In other words, this suggests that the static configuration in classical culture conditions is, at least in part, responsible for the down-regulation of these genes. The designed MCmB could, therefore, be used to study drug metabolism and toxicity in vitro under more physiological conditions for prospective pharmacological or pharmacokinetic studies.

## Abbreviations

AhR: aryl hydrocarbon receptor
CAR: constitutive androstane receptor
CYP: cytochrome P450
FIH: freshly isolated hepatocytes
3MC: 3-methycholanthrene
MCmB: multichamber modular bioreactor
MDR: multidrug resistance protein
PB: phenobarbital
PXR: pregnane X receptor
RIF: rifampicin
UGT: UDP-glucuronosyltransferase
