# Supplementary material for: Modular bioreactor for primary human hepatocyte culture: Medium flow stimulates expression and activity of detoxification genes
Source: Biotechnol J. 2011 May;6(5):554–64. doi: 10.1002/biot.201000326 (PMC3123466; doi:10.1002/biot.201000326)
Supplement: Supplementary file 1 [file biot0006-0554-SD1.pdf]

Table SI.1: Sequence of primers for qRT-PCR

Gene	Sens	Reverse
**AAT**	TGCTGGGGCCATGTTTTTAG	GGGACCAGCTCAACCCTTCT
**AhR**	TGGACAAGGAATTGAAGAAGC	AAAGGAGAGTTTTCTGGAGGAA
**Albumin**	TGCCTGCCTGTTGCCAAAGC	TTGGCAAGGTCCGCCCTGTC
**ApoF**	GGAAGCGATCAAACCTACCA	ATCAGCCTGACAACCAGCTT
**ApoH**	GCACTGAGGAAGGAAAATGG	GGCCATCCAGAGAATATCCA
**ARNT**	TGGCCAAGTCTCGGGTTCCA	TTTGCTGCGCTTGCATCCAT
**CAR**	CCGTGTGGGGTTCCAGGTAG	CAGCCAGCAGGCCTAGCAAC
**C/EBPa**	AGGGACTTGGGGCTTGGAAC	GACCCCCATCGCAGTGAGTT
**CPS1**	TGTCCATTGGTCAGGCTGGA	GCCACCCATGCCCAGAATTA
**CYP1A1**	TCCGGGACATCACAGACAGC	ACCCTGGGGTTCATCACCAA
**CYP1A2**	CATCCCCCACAGCACAACAA	TCCCACTTGGCCAGGACTTC
**CYP2B6**	ATGGGGCACTGAAAAAGACTGA	AGAGGCGGGGACACTGAATGAC
**CYP2C9**	TCCTATCATTGATTACTTCCCG	AACTGCAGTGTTTTCCAAGC
**CYP2D6**	CCTACGCTTCCAAAAGGCTTT	AGAGAACAGGTCAGCCACCCACT
**CYP3A4**	GCCTGGTGCTCCTCTATCTA	GGCTGTTGACCATCATAAAAG
**Coagulation Factor V**	GTGCCCATGACCACATCAGC	CCTCTTCATGTGCCGCCTCT
**Coagulation Factor VII**	TTCAAGGACGCGGAGAGGAC	TGGGGTTTGCTGGCATTTCT
**G6P**	CGTGATCGCAGACCTCAGGA	GGCTCCCTGGTCCAGTCTCA
**GK**	TTCGTGTCGCAGGTGGAGAG	GAAGCTGGGGTGCAGCTTGT
**GR**	CCAACGGTGGCAATGTGAAA	CCGCCAGAGGAGAAAGCAAA
**GSTA**	TGGCAGAGAAGCCCAAGCTC	TGCACCAGCTTCATCCCATC
**HNF4a**	CGCAGATGTGTGTGAGTCCA	CAGTGCCGAGGGACAATGTA
**MDR1**	GACCGGACATCCCAGTGCTT	TGTGCTCGGAGCCACTGAAC
**MRP2**	GGCCCCAATGGCCTGTTGAA	TGGAGGCTGCATCTTCGGGG
**NTCP**	TCACCCTGCCACCCAACTTT	GACCTTGCCCAGCACAAAGG
**OATP8**	GGTCCAGTCATTGGCTTTGC	ACAAGGAAACCAAGCCACCA
**PEPCK 1**	CGGCATCGAGCTGACGGATT	CGCCGTACCCACTGCCAAAG
**PK-L**	ACCGGCAGCTGTTTGAGGAG	CAGAGCGGGTGACAGCAATG
**PXR**	GGACCAGCTGCAGGAGCAAT	CATGAGGGGCGTAGCAAAGG
**RPLP0**	TCGACAATGGCAGCATCTAC	GCCTTGACGTTTTCAGCAAG
**UGT1A1**	GGTGACTGTCCAGGACCTAT	TAGTGGATTTTGGTGAAGGCAGTT
**UGT2B4**	CTTTAGGACTCAATACTCGGCTG	CTCATAGATGCCATTGGCTCCAC
**UGT2B7**	CCTTAGGTCTCAATACTCGGCTG	CTCGTAGATGCCATTGGCTCCA

AAT, α1-antitrypsin; AhR, aryl hydrocarbon receptor; ARNT, aryl hydrocarbon receptor nuclear translocator; Apo, apolipoprotein; CAR, constitutive androstan receptor, C/EBP, CAAT box enhancer binding protein; CPS-1, carbamoyl phosphate synthase-1; CYP, cytochrome P450; G6P, glucose-6-phosphatase; GK, glucokinase; GR, glucocorticoid receptor; GST, glutathion-S-transferase; HNF, hepatocyte nuclear factor; MDR, multidrug resistance; MRP, multidrug resistance protein; NTCP, Na/taurocholate cotransporting polypeptide; OATP, organic anion transporter; PEPCK, phosphoenolpyruvate carboxykinase ; PK-L, pyruvate kinase isoform L; PXR, pregnane X receptor, RPLP0; ribosomal protein, large, P0; UGT, UDP-glucose-glycoprotein glucosyltransferase.
