# Supplementary material for: Modular bioreactor for primary human hepatocyte culture: Medium flow stimulates expression and activity of detoxification genes
Source: Biotechnol J. 2011 May;6(5):554–64. doi: 10.1002/biot.201000326 (PMC3123466; doi:10.1002/biot.201000326)
Supplement: Supplementary file 2 [file biot0006-0554-SD2.pdf]

**Table SI.2. Effect of medium flow on hepatocyte gene expression**

| Gene | Dynamic[a] | | Static[a] | | Sandwich[a] | | Collagen[a] | | FIH | |
|---|---|---|---|---|---|---|---|---|---|---|
| | Percentage | SEM | Percentage | SEM | Percentage | SEM | Percentage | SEM | Percentage | SEM |
| Albumin | 7.89 | 4.29 | 10.16 | 5.61 | 27.57* | 5.71 | 12.19 | 3.96 | 100* | 75.53 |
| AAT | 50.22 | 47.04 | 39.19 | 37.98 | 81.70 | 42.76 | 49.25 | 35.24 | 100 | 54.76 |
| Factor V | 15.43 | 4.54 | 12.74 | 4.78 | 53.99 | 29.55 | 15.45 | 4.61 | 100 | 52.25 |
| Factor VII | 7.03 | 4.48 | 5.78 | 4.53 | 14.02 | 5.95 | 5.90 | 3.53 | 100 | 41.70 |
| CPS1 | 15.31 | 14.98 | 54.26 | 43.14 | 53.60 | 36.73 | 20.30 | 8.67 | 100 | 70.59 |
| G6P | 5.22 | 6.07 | 8.72 | 9.75 | 9.45 | 4.29 | 2.64 | 3.30 | 100* | 53.00 |
| PEPCK 1 | 7.03 | 8.14 | 1.26 | 1.09 | 2.94 | 2.33 | 1.26 | 0.72 | 100 | 27.50 |
| GK | 2.79 | 0.73 | 3.89 | 2.06 | 13.27* | 4.05 | 13.80 | 15.94 | 100 | 63.82 |
| PK-L | 2.66 | 2.08 | 6.86 | 6.43 | 13.74 | 16.45 | 5.61 | 3.78 | 100 | 49.75 |
| ApoF | 0.77 | 0.71 | 2.49 | 2.30 | 4.84 | 2.42 | 2.14 | 1.85 | 100 | 61.70 |
| ApoH | 2.56 | 1.62 | 4.48 | 3.12 | 7.83 | 4.99 | 5.70 | 3.02 | 100* | 42.05 |
| HNF4a | 82.51 | 23.73 | 70.16 | 20.95 | 97.03 | 42.76 | 78.14 | 28.45 | 100 | 61.81 |
| C/EBPa | 29.19 | 9.39 | 20.97 | 10.45 | 81.83 | 25.25 | 33.15 | 19.65 | 100 | 55.15 |

NQ: Not Quantifiable NC: Not Calculable

*$p<0.05$ with respect to gene expression in dynamic conditions (n=6)
a. Dynamic and Static: see Fig 2. Sandwich and Collagen: standard conditions in collagen sandwich configuration or on collagen ($0.8 \times 10^6$ cells per ml). See Experimental for detail.
