# Supplementary material for: Modular bioreactor for primary human hepatocyte culture: Medium flow stimulates expression and activity of detoxification genes
Source: Biotechnol J. 2011 May;6(5):554–64. doi: 10.1002/biot.201000326 (PMC3123466; doi:10.1002/biot.201000326)
Supplement: Supplementary file 3 [file biot0006-0554-SD3.pdf]

Table SI.3. Effect of medium flow on albumin and urea production

Production of	Dynamic	Static
Albumin[a]	15±6	11±6
Urea[b]	3.2±0.1	3.1±0.1

a: Albumin protein production in the MCmB (dynamic) *versus* static culture medium, in ng/mL for the same number of cells. Values presented are average±SD of three experiments carried out with three different hepatocyte preparations from different donors (FT297, FT298 and FT301)

b: Urea production in the MCmB (dynamic) *versus* static culture medium, in ng/mL for the same number of cells. Values presented are average±SD of three experiments carried out with three different hepatocyte preparations from different donors (FT297, FT298 and FT301)
